# Supplementary material for: p53 regulates diverse tissue-specific outcomes to endogenous DNA damage in mice
Source: Nat Commun. 2024 Mar 21;15:2518. doi: 10.1038/s41467-024-46844-1 (PMC10957910; doi:10.1038/s41467-024-46844-1)
Supplement: Supplementary file 1 — Supplementary Information [file 41467_2024_46844_MOESM1_ESM.pdf]

Supplementary Information

p53 regulates diverse tissue-specific outcomes to endogenous DNA damage in mice

Ross J. Hill[1], Nazareno Bona[1], Job Smink[2], Hannah Webb[1], Alastair Crisp[1], Juan I. Garaycoechea[2*], Gerry P. Crossan[1*]

[1] MRC Laboratory of Molecular Biology, Cambridge Biomedical Campus, Francis Crick Avenue, Cambridge, United Kingdom
[2] Hubrecht Institute, Royal Netherlands Academy of Arts and Sciences (KNAW), Utrecht, the Netherlands

* Correspondence gcrossan@mrc-lmb.cam.ac.uk or juan.g@hubrecht.eu

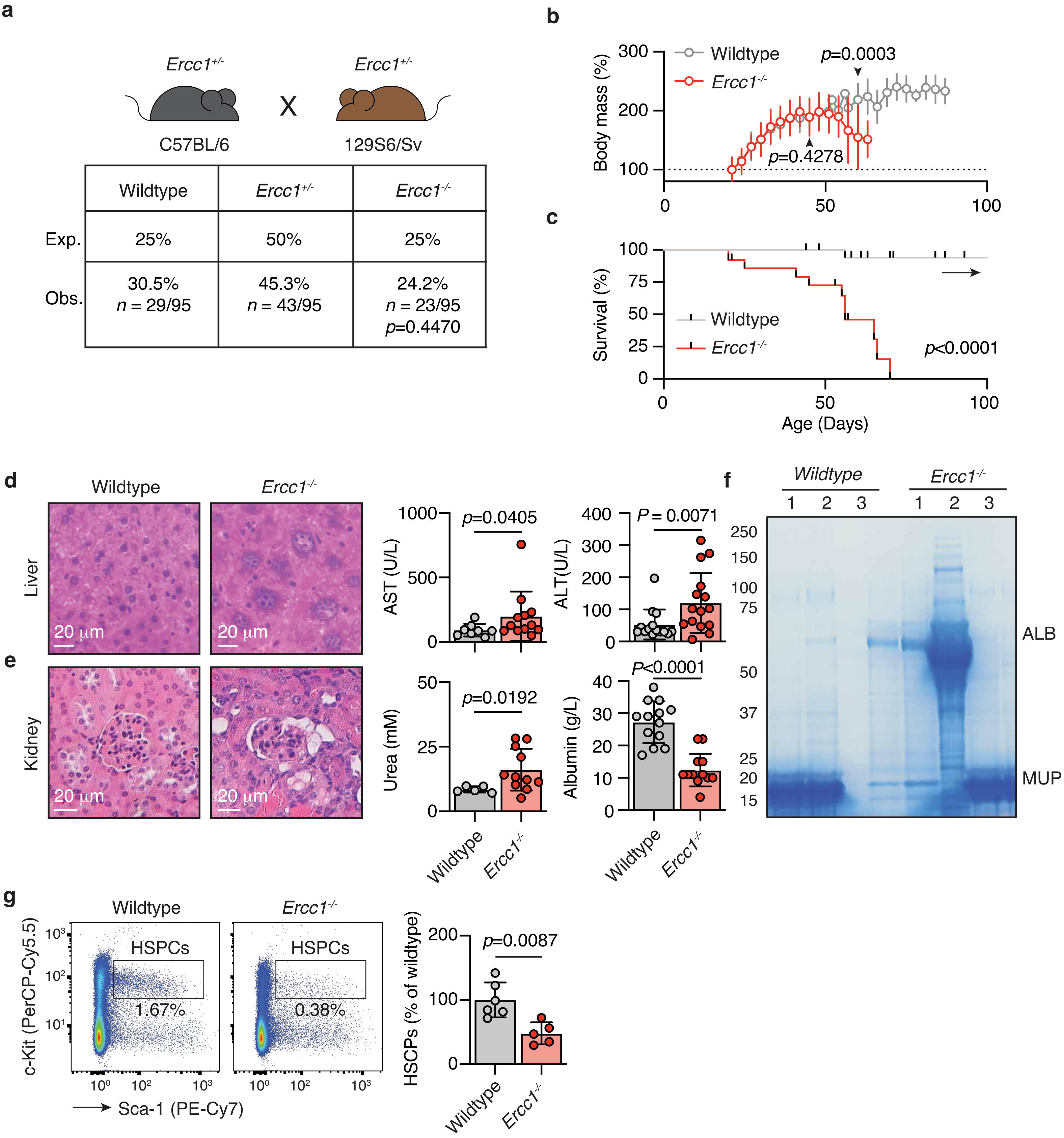

Supplementary Figure 1 - Characterization of *Ercc1-/-* allele

Supplementary Figure 1 – Characterization of *Ercc1$^{-/-}$* allele. **a**, *Ercc1$^{-/-}$* mice are observed at expected Mendelian ratios at postnatal day 21 (*p-value* calculated by one tailed Chi-square test). **b**, Growth of *Ercc1$^{-/-}$* and wildtype littermate controls based on daily weights (*p*-values calculated by two-tailed Mann-Whitney *U*-test, data are mean ± s.d.; *n* = 16 Wildtype and 19 *Ercc1$^{-/-}$* mice). **c**, Kaplan-Meier survival curve showing the survival of cohorts of *Ercc1$^{-/-}$* and wildtype littermate controls (*p*-value calculated by one tailed Mantel-Cox test, *n* = 29 Wildtype and 23 *Ercc1$^{-/-}$* mice). **d**, Representative H&E-stained sections of liver and terminal serum aspartate transaminase (AST) and alanine transaminase (ALT) levels from 6-to-8-week-old *Ercc1$^{-/-}$* and control mice (*p*-value calculated by two-tailed Mann-Whitney *U*-test, data are mean ± s.d.; *n* = 8 and 12 mice, left to right.). **e**, Representative H&E-stained sections of kidney and terminal serum urea and albumin levels from 6-to-8-week-old *Ercc1$^{-/-}$* and control mice (*p*-value calculated by two-tailed Mann-Whitney *U*-test, data are mean ± s.d.; *n* = 5 and 11 mice, left to right). **f,** Representative Coomasie gel of urine from wildtype or 6-to-8-week-old *Ercc1$^{-/-}$* mice each lane represents one mouse, ALB-albumin, MUP-major urinary proteins **g**, Quantification of HSPCs (lineage$^-$ CD41$^-$ Sca-1$^+$ c-Kit$^+$) by flow cytometry from wildtype and *Ercc1$^{-/-}$* adult mice (*p*-value calculated by two-tailed Mann-Whitney *U*-test, data are mean ± s.d.; *n* = 6 Wildtype and 5 *Ercc1$^{-/-}$* mice).

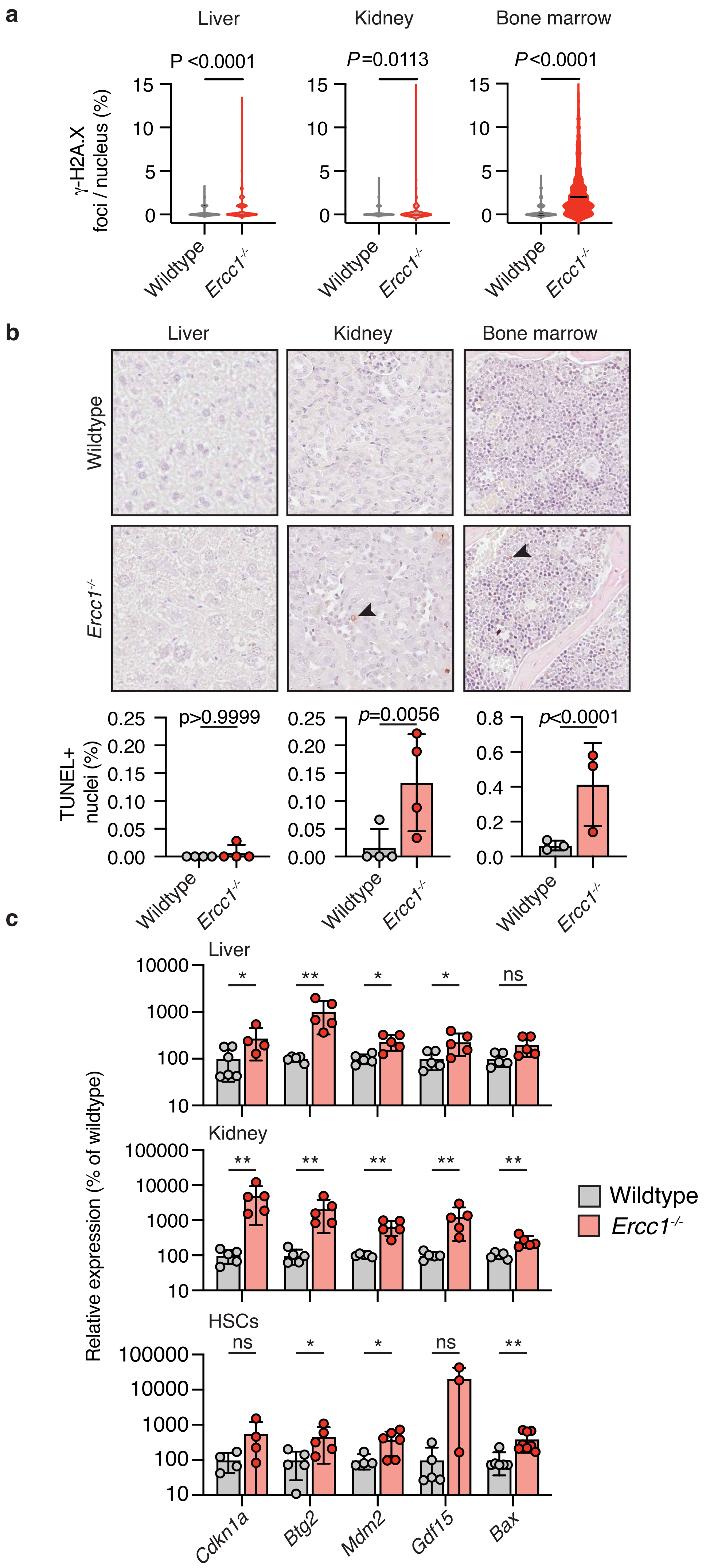

Supplementary Figure 2 - DNA damage, apoptosis, and transcriptional changes in Ercc1-/-

Supplementary Figure 2 – RT-qPCR data in wildtype and *Ercc1*$^{-/-}$ mice. a, Quantitative of the frequency of γ-H2A.X foci per nucleus. For each genotype 3 independent mice were assessed, *p*-value calculated by two-tailed t-test, data are median; *n* =327, 327, 770, 645, 299, 299, left to right **b,** Representative images of TUNEL assay in liver, kidney and bone marrow of wildtype or *Ercc1*$^{-/-}$ mice (arrowheads indicate TUNEL^{+} nuclei) and quantification of the frequency of TUNEL^{+} nuclei. *p*-value calculated by two-tailed Mann-Whitney *U*-test, data are mean ± s.d.; *n* = 4, 4, 4, 4, 3 and 3 mice, left to right) **c,** RT-qPCR expression analysis of the p53 target genes (*Cdkn1a*, *Btg2*, *Mdm2*, *Gdf15* and *Bax*) in the liver, kidney and HSCs of wildtype and *Ercc1*$^{-/-}$ mice (*p*-values calculated by two-tailed Mann-Whitney *U*-test; *$p<0.05$, ** $p<0.01$, data are mean ± s.d.; *n* = liver (6, 4, 5, 5, 5, 5, 5, 5, 5 and 5), Kidney (5, 5, 5, 5, 5, 5, 4, 5, 5 and 5) and HSCs (4, 4, 5, 5, 4, 6, 5, 3, 6 and 7) mice, left to right).

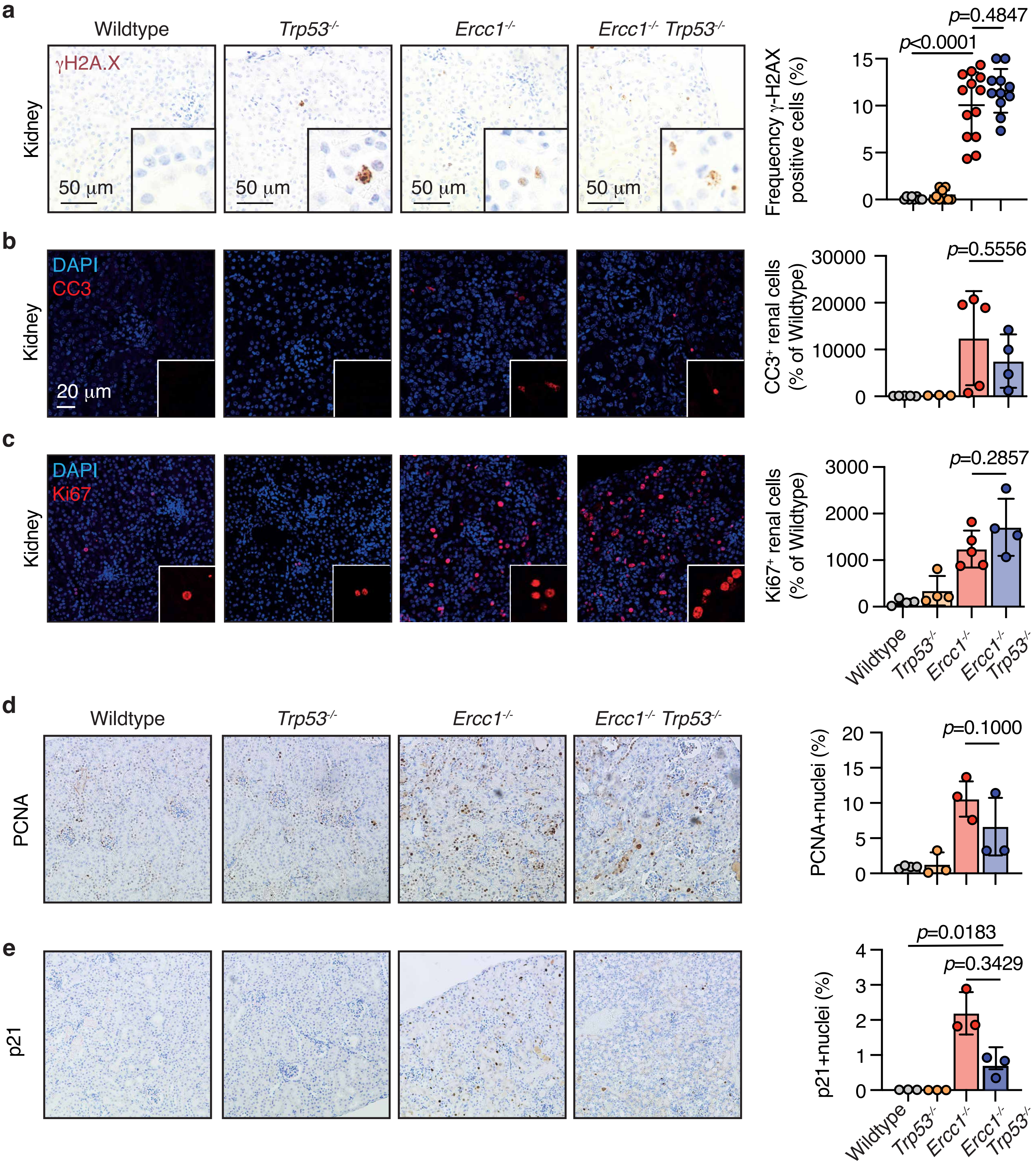

Supplementary Figure 3 - p53 is not involved in kidney pathogenesis in the absence of ERCC1

Supplementary Figure 3 – p53 is not involved in kidney pathogenesis in the absence of ERCC1. **a**, Representative images of γ-H2A.X foci in kidney sections from wildtype and *Ercc1*$^{-/-}$ *Trp53*$^{-/-}$ mice and quantification of the frequency of cells with >5 nuclear γ-H2A.X foci (*p*-values calculated by two-tailed Mann-Whitney *U*-test, data are mean ± s.d.; each dot corresponds to individual mice. n= 8, 10, 13, 11 left to right) **b**, Representative immunofluorescence of cleaved caspase 3 (CC3) staining in the kidney of *Ercc1*$^{-/-}$ *Trp53*$^{-/-}$ and control mice and quantification of the frequency of CC3^{+} renal cells in the kidney of *Ercc1*$^{-/-}$ *Trp53*$^{-/-}$ and control mice (*p*-value calculated by two-tailed Mann-Whitney *U*-test, data are mean ± s.d.; *n* = 5, 3, 5 and 4 independent mice, left to right). **c**, Representative immunofluorescence of Ki67 staining in the kidney of 6-to-8-week-old *Ercc1*$^{-/-}$ *Trp53*$^{-/-}$ and control mice. **c**, Representative immunofluorescence of Ki67 staining in the kidney of *Ercc1*$^{-/-}$ *Trp53*$^{-/-}$ and control mice and quantification of the frequency of Ki67^{+} renal cells in the kidney of 6-to-8-week-old *Ercc1*$^{-/-}$ *Trp53*$^{-/-}$ and control mice (*p*-value calculated by two-tailed Mann-Whitney *U*-test, data are mean ± s.d.; *n* = 4, 4, 5 and 4 independent mice, left to right). **d,** Representative immunohistochemistry of PCNA staining in the kidney of *Ercc1*$^{-/-}$ *Trp53*$^{-/-}$ and control mice and quantification of the frequency of PCNA^{+} renal cells in the kidney of *Ercc1*$^{-/-}$ *Trp53*$^{-/-}$ and control mice (*p*-value calculated by two-tailed Mann-Whitney *U*-test, data are mean ± s.d.; *n* = 3 independent mice). **e,** Representative immunohistochemistry of p21 staining in the kidney of *Ercc1*$^{-/-}$ *Trp53*$^{-/-}$ and control mice and quantification of the frequency of p21^{+} renal cells in the kidney of *Ercc1*$^{-/-}$ *Trp53*$^{-/-}$ and control mice (*p*-value calculated by two-tailed Mann-Whitney *U*-test, data are mean ± s.d.; *n* = 3 independent mice).

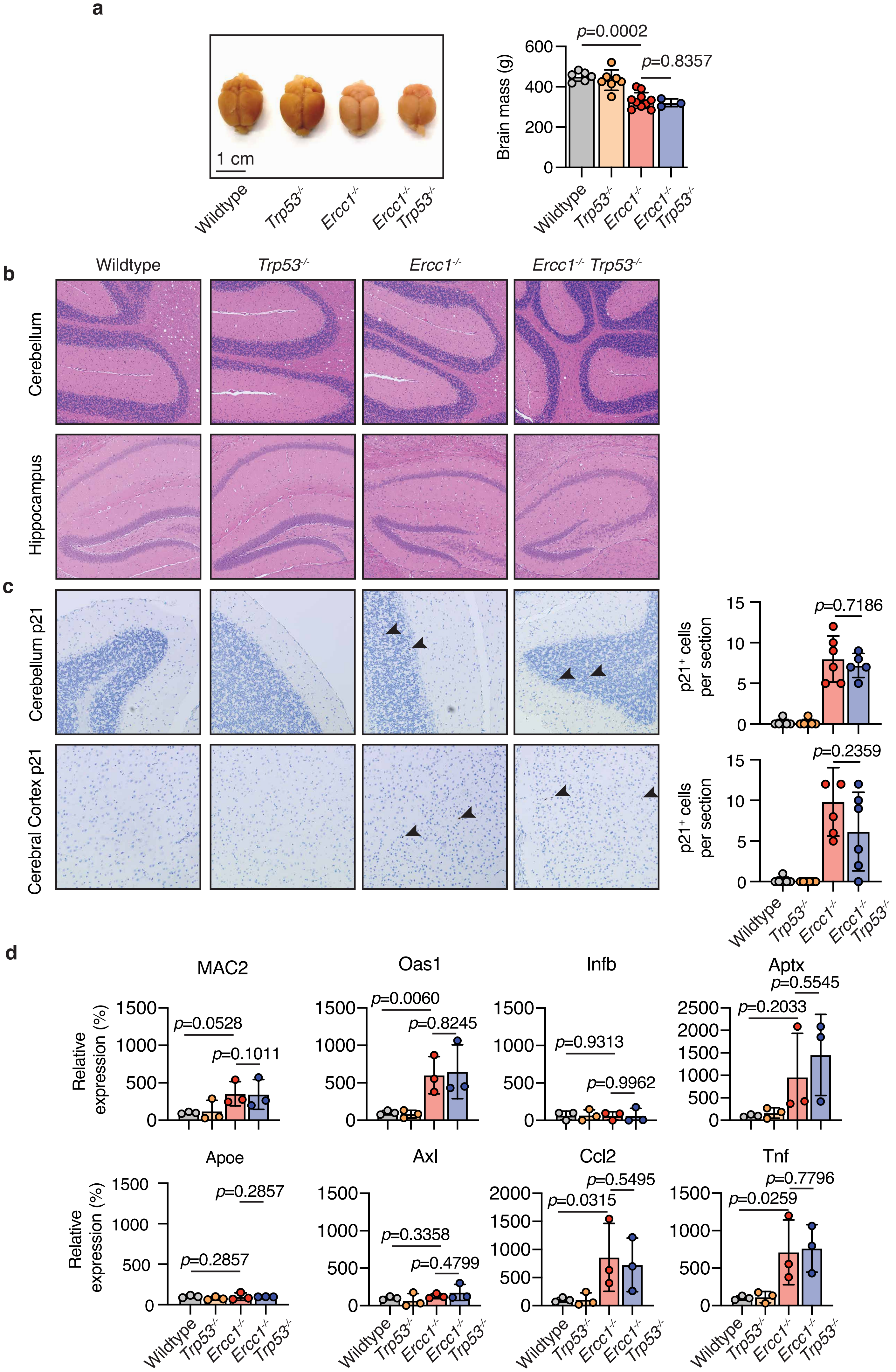

Supplementary Figure 4 - p53 does not drive neuropathology in *Ercc1*-/- mice

Supplementary Figure 4 – p53 does not drive neuropathology in *Ercc1$^{-/-}$* mice. a, Representative images of brains from age-matched adult *Ercc1$^{-/-}$ Trp53$^{-/-}$* and control mice and quantification brain weight (*p*-values calculated by two-tailed Mann-Whitney *U*-test, data are mean ± s.d.; *n* = 6 Wildtype, 7 *Trp53$^{-/-}$,* 10 *Ercc1$^{-/-}$* and 3 *Ercc1$^{-/-}$ Trp53$^{-/-}$* independent mice). **b**, Representative H&E-stained sections of the cerebellum (top) and hippocampus (bottom) of 6-to-8-week-old *Ercc1$^{-/-}$ Trp53$^{-/-}$* and control mice. **c,** Representative immunohistochemistry of p21 staining in the brain of *Ercc1$^{-/-}$ Trp53$^{-/-}$* and control mice (arrowheads indicate p21^{+} nuclei) and quantification of the frequency of p21^{+} cells in the brain of *Ercc1$^{-/-}$ Trp53$^{-/-}$* and control mice (*p*-value calculated by two-tailed Mann-Whitney *U*-test, data are mean ± s.d.; *n* = 3 independent mice per genotype). **d,** RT-PCR expression analysis of inflammatory markers in the whole brain (*p*-values calculated by two-tailed Mann-Whitney *U*-test, data are mean ± s.d, n= 3 independent mice per condition).

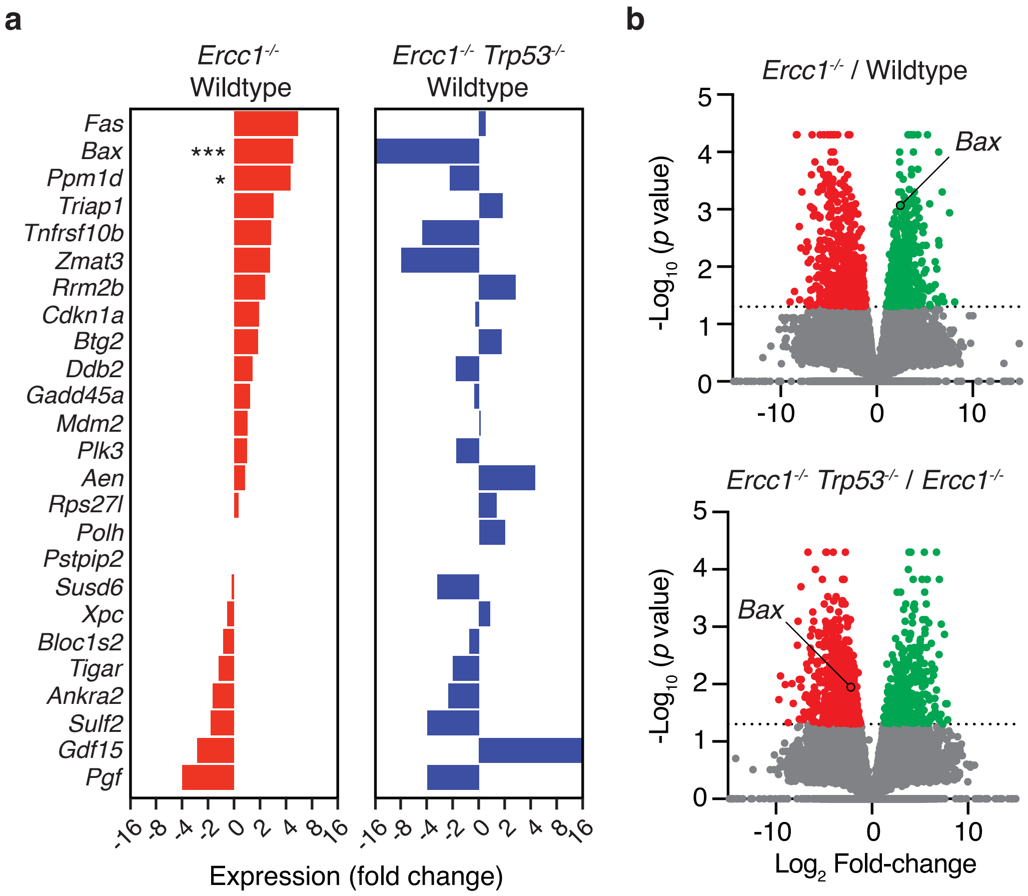

Supplementary Figure 5 - p53 deletion rescues blood cell apoptosis in *Ercc1*$^{-/-}$ mice

Supplementary Figure 5 – p53 deletion rescues blood cell apoptosis in *Ercc1*$^{-/-}$ mice. a, Expression analysis of a p53 signature comprising the top 25 p53 target genes in adult *Ercc1*$^{-/-}$ (left) and *Ercc1*$^{-/-}$ *Trp53*$^{-/-}$ (right) HSCs relative to wildtype controls (*p*-values calculated by two-tailed Mann-Whitney *U*-test; **p*<0.05, ** *p*<0.01, *p*<0.001, data represent mean and s.d.; *n* = 3 independent mice per genotype). **b**, Volcano plots showing the differentially expressed genes in adult HSCs from global transcriptome data; (top) *Ercc1*$^{-/-}$ compared to wildtype controls (bottom) *Ercc1*$^{-/-}$ *Trp53*$^{-/-}$ compared with *Ercc1*$^{-/-}$ controls.

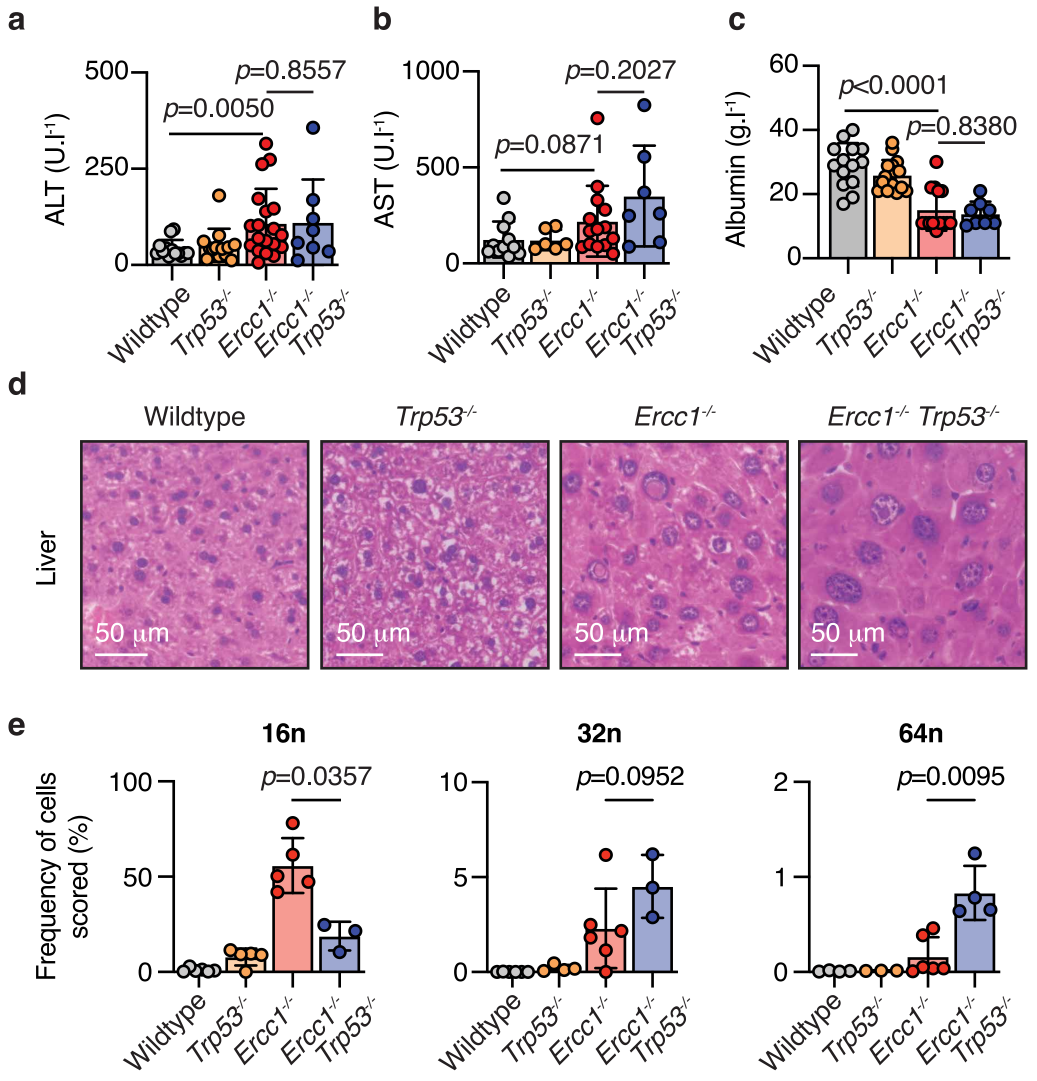

Supplementary Figure 6 - p53 limits liver polyploidisation in the absence of ERCC1 but does not protect liver function

Supplementary Figure 6 – p53 limits liver polyploidisation in the absence of ERCC1 but does not protect liver function. **a**, Terminal blood serum alanine transaminase (ALT) levels from *Ercc1*$^{-/-}$ *Trp53*$^{-/-}$ and control mice (*p*-value calculated by two-tailed Mann-Whitney *U*-test, data are mean ± s.d.; *n* = 10, 7, 10 and 6 independent mice, left to right). **b**, Terminal blood serum aspartate transaminase (AST) levels from *Ercc1*$^{-/-}$ *Trp53*$^{-/-}$ and control mice (*p*-value calculated by two-tailed Mann-Whitney *U*-test, data are mean ± s.d.; *n* = 10, 5, 10 and 6 independent mice, left to right). **c**, Terminal blood serum albumin levels from *Ercc1*$^{-/-}$ *Trp53*$^{-/-}$ and control mice (*p*-value calculated by two-tailed Mann-Whitney *U*-test, data are mean ± s.d.; *n* = 9, 9, 7 and 4 independent mice, left to right). **d**, H&E-stained sections of liver from 6-to-8-week-old *Ercc1*$^{-/-}$ *Trp53*$^{-/-}$ and control mice. **e**, Quantification of liver cell nuclei with chromosome complements of 16n (left), 32n (centre) and 64n (right) from the liver of *Ercc1*$^{-/-}$ *Trp53*$^{-/-}$ and control mice by flow cytometry (*p*-values calculated by two-tailed Mann-Whitney *U*-test, data are mean ± s.d.; *n* = 16n; 6, 5, 5 and 3, 32n; 6, 4, 6 and 3, 64n; 4, 3, 6 and 4, independent mice, left to right).

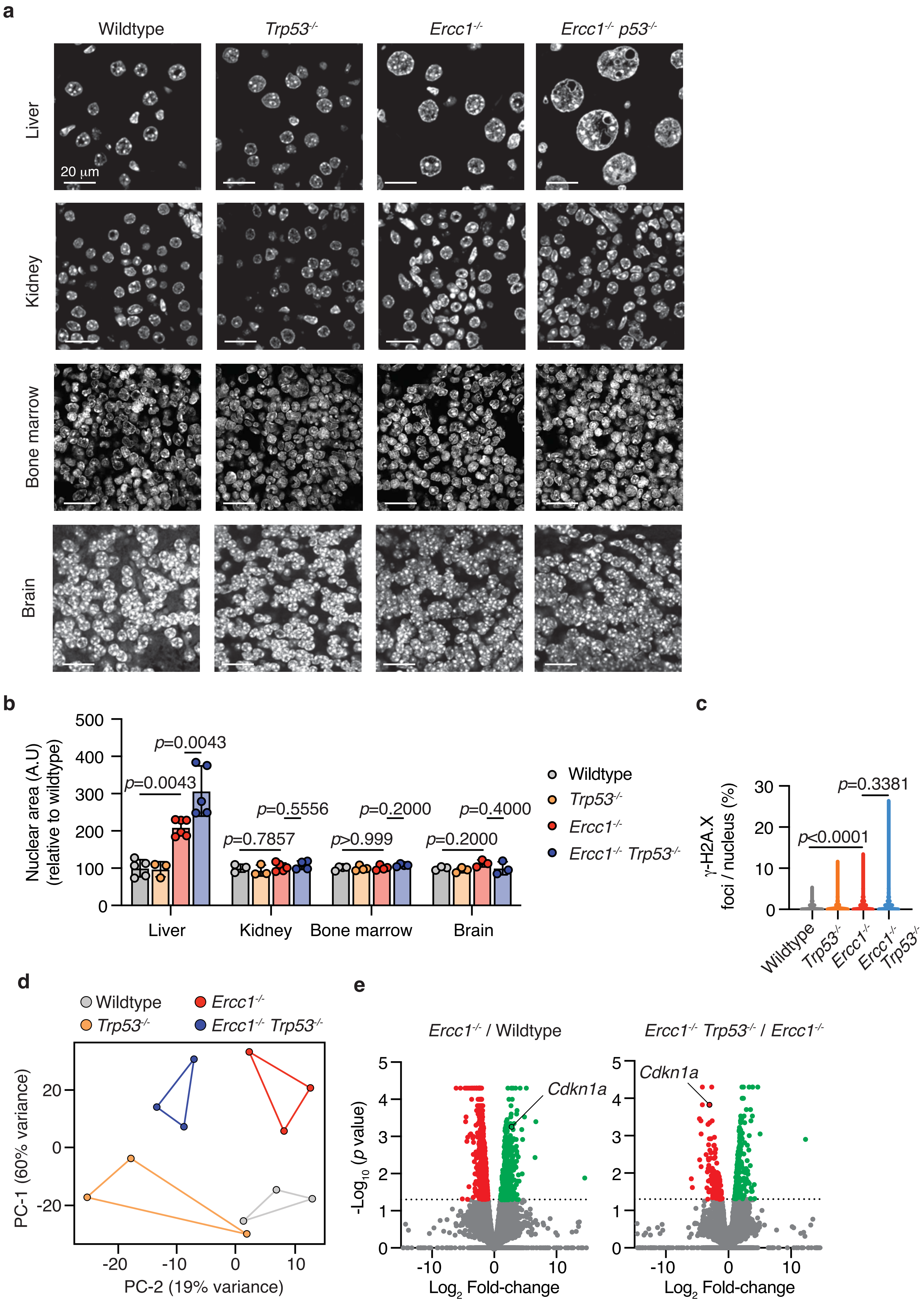

Supplementary Figure 7 - Nuclear area in different tissues from wildtype and mutant mice and RNAseq data in hepatocytes

Supplementary Figure 7 – Nuclear area in different tissues from wildtype and mutant mice and RNAseq data in hepatocytes. a, Representative immunofluorescence of DAPI staining in the liver, kidney, bone marrow and brain of 6-to-8-week-old *Ercc1*$^{-/-}$ *Trp53*$^{-/-}$ and control mice. **b**, Quantification of the nuclear area in the liver, kidney, bone marrow and cerebral cortex of 6-to-8-week-old *Ercc1*$^{-/-}$ *Trp53*$^{-/-}$ and control mice (*p*-value calculated by two-tailed Mann-Whitney *U*-test, data are mean ± s.d.; each dot corresponds to an independent mouse, n=5, 3, 6, 5, 3, 3, 4, 5, 4, 4, 4, 4, 3, 3, 3, 3). **c**, Quantitative of the frequency of γ-H2A.X foci per nucleus. For each genotype 100 nuclei were quantified in 3 mice. **d**, Volcano plots showing the differentially expressed genes in adult hepatocytes from global transcriptome data; (left) *Ercc1*$^{-/-}$ compared with wildtype controls, (right) *Ercc1*$^{-/-}$ *Trp53*$^{-/-}$ compared with *Ercc1*$^{-/-}$ controls. **e**, Principal component analysis (PCA) showing changes associated with loss of *Trp53* and/or *Ercc1* in adult hepatocytes based on global transcriptome data (data represent 3 independent mice per genotype).

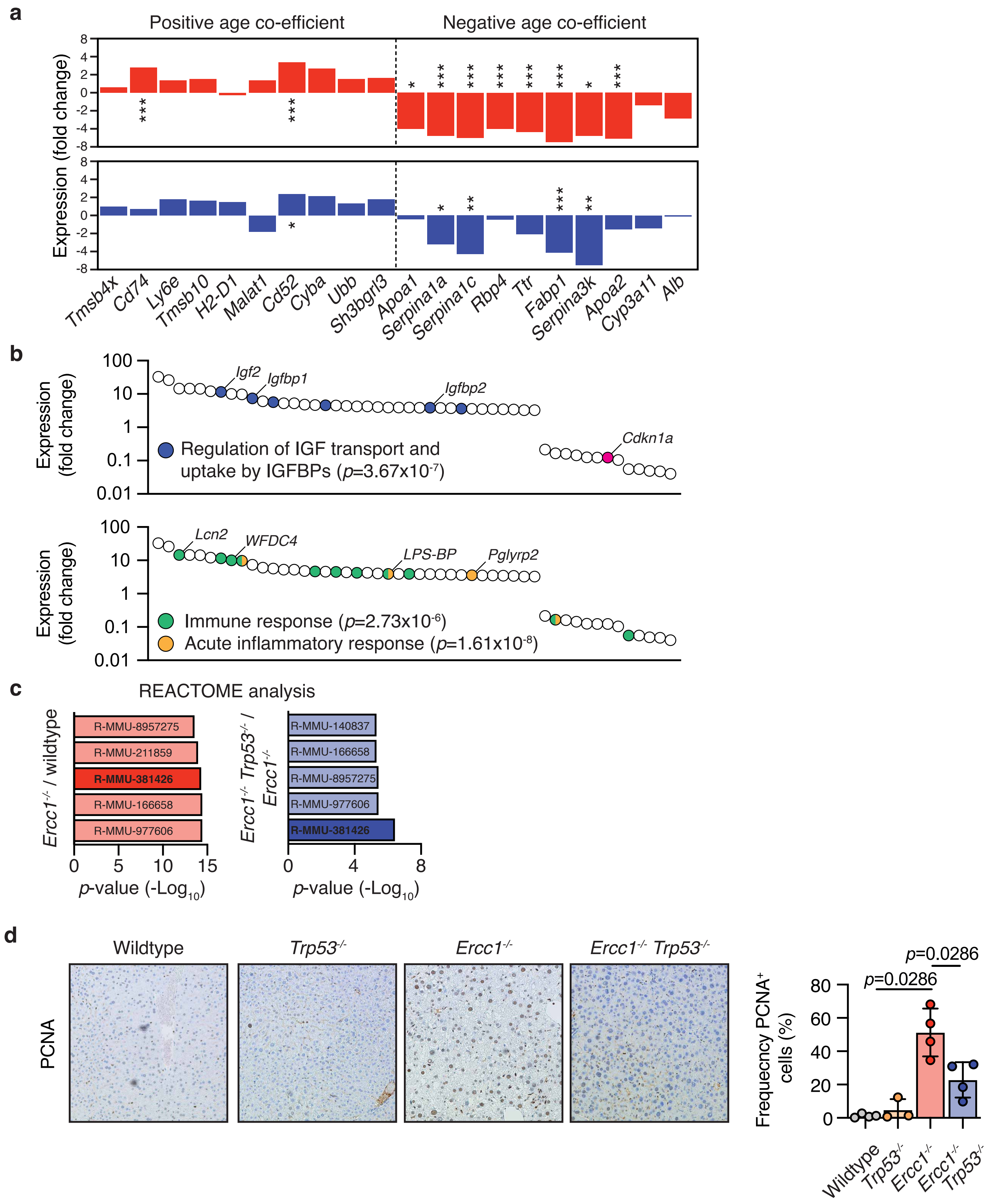

Supplementary Figure 8 - p53 loss exacerbates features of ageing in the liver of *Ercc1*$^{-/-}$ mice

Supplementary Figure 8 – p53 loss exacerbates features of aging in the liver of *Ercc1*$^{-/-}$ mice. a, Expression analysis of the Mouse Aging Cell Atlas liver aging signature (*Tabula Muris Senis*) in *Ercc1*$^{-/-}$ (left) and *Ercc1*$^{-/-}$ *Trp53*$^{-/-}$ (right) hepatocytes each made relative to wildtype controls from global transcriptome data (*p*-values calculated by two-tailed Mann-Whitney *U*-test; **p*<0.05, ** *p*<0.01, *p*<0.001, data are mean; *n* = 3 per genotype). **b**, Representative plots of the top 50 most significantly differentially expressed genes in *Ercc1*$^{-/-}$ *Trp53*$^{-/-}$ hepatocytes relative to *Ercc1*$^{-/-}$ controls identifying (top) components of the "Regulation of IGF transport and uptake by IGFBPs" (bottom) factors involved in "Immune response" and "Acute inflammatory response". **c**, Pathway enrichment using the REACTOME database of the differentially expressed genes in *Ercc1*$^{-/-}$ and *Ercc1*$^{-/-}$ *Trp53*$^{-/-}$ hepatocytes relative to wildtype controls from global transcriptome data (*n* = 3 per genotype). **d,** Representative immunohistochemistry of PCNA staining in the liver of *Ercc1*$^{-/-}$ *Trp53*$^{-/-}$ and control mice and quantification of the frequency of PCNA^{+} hepatocytes in the liver of *Ercc1*$^{-/-}$ *Trp53*$^{-/-}$ and control mice (*p*-value calculated by two-tailed Mann-Whitney *U*-test, data are mean ± s.d.; each dot corresponds to an independent mouse, n= 4, 3, 3, 4)

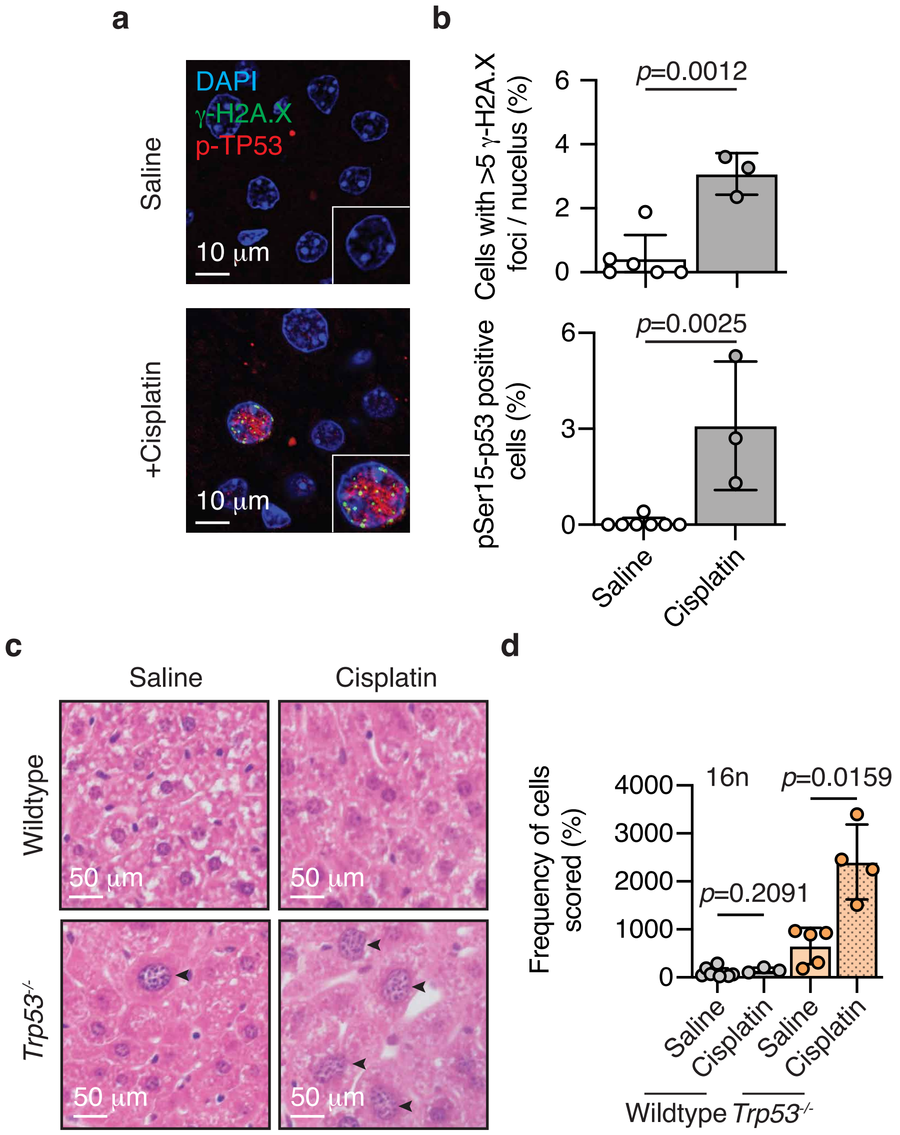

Supplementary Figure 9 - The p53-axis protects the liver from chemotherapy induced polyploidisation

Supplementary Figure 9 – The p53-axis protects the liver from chemotherapy-induced polyploidisation. **a**, Representative images of phosphorylated p53 (pSer15-TP53) and γ-H2A.X foci in the liver of wildtype mice exposed to 4 $mg.kg^{-1}$ cisplatin or vehicle-only control and quantification of the frequency of pSer15-TP53^{+} cells (*p*-values calculated by two-tailed Mann-Whitney *U*-test, data are mean ± s.d.; *n* = liver; 7 and 3 independent mice, left to right). **b**, Quantification of the frequency of cells with >5 nuclear γ-H2A.X foci (*p*-values calculated by two-tailed Mann-Whitney *U*-test, data are mean ± s.d.; *n* = liver; 6 and 3, kidney; 6 and 3 independent mice, left to right); and the frequency of pSer15-TP53^{+} cells (*p*-values calculated by two-tailed Mann-Whitney *U*-test, data are mean ± s.d.; *n* = liver; 7 and 3, kidney; 7 and 3 independent mice, left to right). **c**, H&E-stained sections of liver from wildtype and *Trp53*$^{-/-}$ mice exposed to 4 $mg.kg^{-1}$ cisplatin or vehicle-only control, arrowheads indicate polyploidy hepatocyte nuclei. **d**, Quantification of liver cell nuclei with chromosome complements of 16n from the liver wildtype and *Trp53*$^{-/-}$ mice exposed to 4 $mg.kg^{-1}$ cisplatin or vehicle-only controls (*p*-values calculated by two-tailed Mann-Whitney *U*-test, data are mean ± s.d.; *n* = 16n; 9, 3, 5 and 4, 32n; 9, 3, 6 and 4 independent mice, left to right).

a

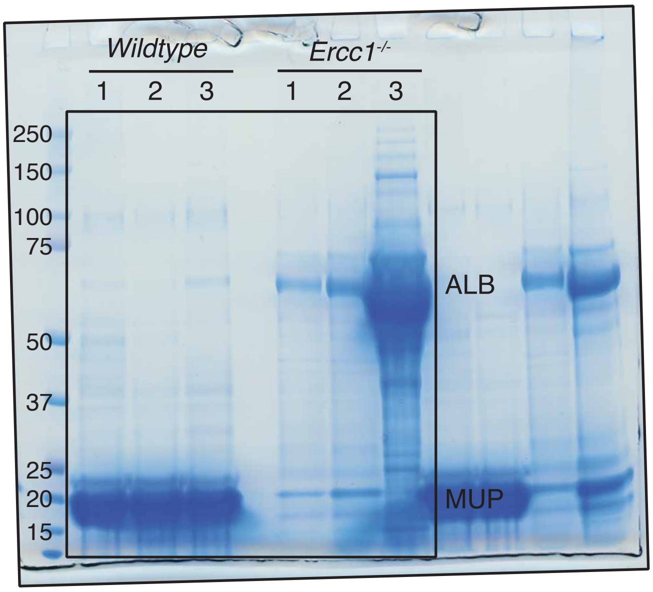

Uncropped gel from Supplementary Fig. 1f

Supplementary Figure 10 - Uncropped Gels

Supplementary Figure 10 – Uncropped gels. a, Uncropped gel from Supplementary Figure 1f

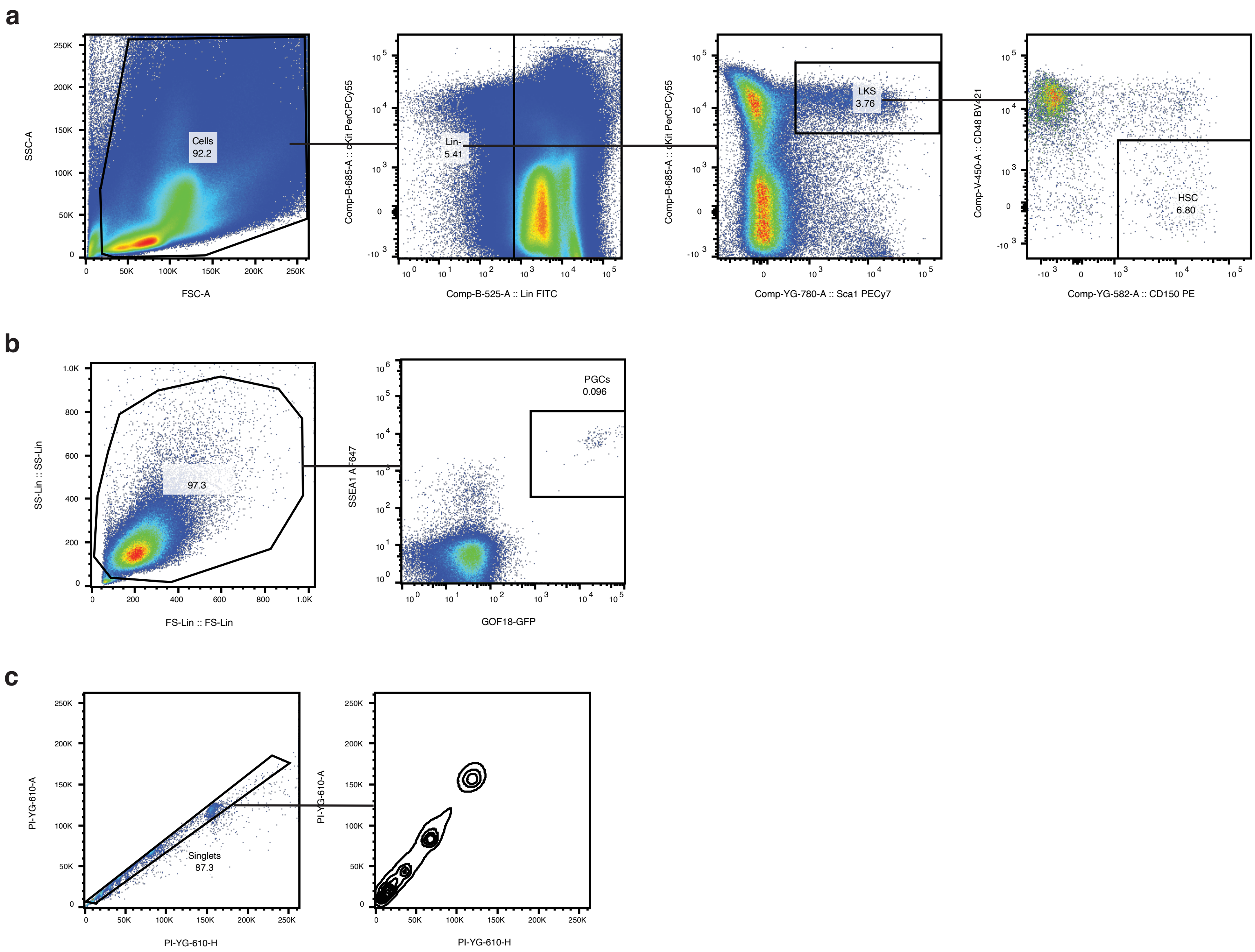

Supplementary Figure 11 - Gating Strategy

Supplementary Figure 11 – Flow cytometry gating strategies. a, Gating strategy for analysis of HSPCs (LKS) and HSCs. **b,** Gating strategy for quantification of PGCs. **C,** Gating strategy for the quantification of polypoid nuclei.
