# Supplementary material for: p53 regulates diverse tissue-specific outcomes to endogenous DNA damage in mice
Source: Nat Commun. 2024 Mar 21;15:2518. doi: 10.1038/s41467-024-46844-1 (PMC10957910; doi:10.1038/s41467-024-46844-1)
Supplement: Supplementary file 3 — Description of Additional Supplementary Files [file 41467_2024_46844_MOESM3_ESM.pdf]

Description of Additional Supplementary Files

File Name: Supplementary Data 1
Description: Table of the oligonucleotide sequences used in this study.
