# Supplementary material for: p53 regulates diverse tissue-specific outcomes to endogenous DNA damage in mice
Source: Nat Commun. 2024 Mar 21;15:2518. doi: 10.1038/s41467-024-46844-1 (PMC10957910; doi:10.1038/s41467-024-46844-1)
Supplement: Supplementary file 5 — Reporting Summary [file 41467_2024_46844_MOESM5_ESM.pdf]

Corresponding author(s): Crossan and Garayceochea

Last updated by author(s): 21 Feb 2024

Reporting Summary

Nature Portfolio wishes to improve the reproducibility of the work that we publish. This form provides structure for consistency and transparency in reporting. For further information on Nature Portfolio policies, see our Editorial Policies and the Editorial Policy Checklist.

Statistics

For all statistical analyses, confirm that the following items are present in the figure legend, table legend, main text, or Methods section.

n/a	Confirmed	
☐	☒	The exact sample size (*n*) for each experimental group/condition, given as a discrete number and unit of measurement
☐	☒	A statement on whether measurements were taken from distinct samples or whether the same sample was measured repeatedly
☐	☒	The statistical test(s) used AND whether they are one- or two-sided<br>*Only common tests should be described solely by name; describe more complex techniques in the Methods section.*
☒	☐	A description of all covariates tested
☒	☐	A description of any assumptions or corrections, such as tests of normality and adjustment for multiple comparisons
☐	☒	A full description of the statistical parameters including central tendency (e.g. means) or other basic estimates (e.g. regression coefficient) AND variation (e.g. standard deviation) or associated estimates of uncertainty (e.g. confidence intervals)
☒	☐	For null hypothesis testing, the test statistic (e.g. *F*, *t*, *r*) with confidence intervals, effect sizes, degrees of freedom and *P* value noted<br>*Give P values as exact values whenever suitable.*
☒	☐	For Bayesian analysis, information on the choice of priors and Markov chain Monte Carlo settings
☒	☐	For hierarchical and complex designs, identification of the appropriate level for tests and full reporting of outcomes
☒	☐	Estimates of effect sizes (e.g. Cohen's *d*, Pearson's *r*), indicating how they were calculated

Our web collection on statistics for biologists contains articles on many of the points above.

Software and code

Policy information about availability of computer code

Data collection	Data was collected using FACSDiva (BD) Software v6.2 on LSRII FACS analyser (BD Pharmingen) or ECLIPSE analyser (Sony Biotechnology) RNA sequencing was performed onNovaSeq 6000 system (Illumina)
Data analysis	Data was analyzed using Graphpad prism v7. Flow cytometry data was analyzed using FlowJo v10.1r5. The open-source software, tools, and packages used for data analysis in this study, as well as the version of each program, were trim_galore (v0.4.4), hisat (v2-2.2.1), cuffdiff (v2.2.1).

For manuscripts utilizing custom algorithms or software that are central to the research but not yet described in published literature, software must be made available to editors and reviewers. We strongly encourage code deposition in a community repository (e.g. GitHub). See the Nature Portfolio guidelines for submitting code & software for further information.

Data

Policy information about availability of data

All manuscripts must include a data availability statement. This statement should provide the following information, where applicable:

- Accession codes, unique identifiers, or web links for publicly available datasets
- A description of any restrictions on data availability
- For clinical datasets or third party data, please ensure that the statement adheres to our policy

All data supporting the findings of this study are available within the paper and Supplementary information and Source data. The RNA-sequencing data generated in

this study have been deposited in the NCBI Gene Expression Omnibus database under accession code GSE206778 [https://www.ncbi.nlm.nih.gov/geo/query/acc.cgi?acc=GSE206778].

Research involving human participants, their data, or biological material

Policy information about studies with human participants or human data. See also policy information about sex, gender (identity/presentation), and sexual orientation and race, ethnicity and racism.

Reporting on sex and gender	N/A
Reporting on race, ethnicity, or other socially relevant groupings	N/A
Population characteristics	N/A
Recruitment	N/A
Ethics oversight	N/A

Note that full information on the approval of the study protocol must also be provided in the manuscript.

Field-specific reporting

Please select the one below that is the best fit for your research. If you are not sure, read the appropriate sections before making your selection.

☒ Life sciences ☐ Behavioural & social sciences ☐ Ecological, evolutionary & environmental sciences

For a reference copy of the document with all sections, see nature.com/documents/nr-reporting-summary-flat.pdf

Life sciences study design

All studies must disclose on these points even when the disclosure is negative.

Sample size	For the analysis of Mendelian segregation of alleles, samples size was determined by power analysis using the following site http://biomath.info/power/chsq1gp.htm. Sufficient animals were used in order to detect a 50% reduction in expected frequency, using power of 0.8 and alpha 0.05. Others samples sizes were not predetermined as the magnitude of effect was unknown prior to the experiment. Others samples sizes were based on previously published results : PGC quantification PMID:31367016,25010009 ; DNA damage assessment PMID:31367016,9488723, Ercc1 phenotyping PMID:22953029, 8275084, 17183314
Data exclusions	No data were excluded from analyses.
Replication	All experiments were repeated as described in the figure legends or materials and methods but on all occasions each experiment was performed at least three times. Different biological replicates were performed on separated days, with samples collected from independent biological animals on different days and processed on different days. Samples were allocated to groups based upon subsequent genotyping. Experiments were reproducible across all repeats. All replication data are included and confirm the original findings.
Randomization	No randomization was involved as groups are based upon the genotype of the animal/emrbyo.
Blinding	The investigators were blind to the genotypes of mice and relied solely on identification numbers throughout the study.

Reporting for specific materials, systems and methods

We require information from authors about some types of materials, experimental systems and methods used in many studies. Here, indicate whether each material, system or method listed is relevant to your study. If you are not sure if a list item applies to your research, read the appropriate section before selecting a response.

Materials & experimental systems

n/a	Involved in the study
☐	☒ Antibodies
☒	☐ Eukaryotic cell lines
☒	☐ Palaeontology and archaeology
☐	☒ Animals and other organisms
☒	☐ Clinical data
☒	☐ Dual use research of concern
☒	☐ Plants

Methods

n/a	Involved in the study
☒	☐ ChIP-seq
☐	☒ Flow cytometry
☒	☐ MRI-based neuroimaging

Antibodies

Antibodies used

anti-CD4 (1:100 clone H129.19, BD Pharmingen), CD3e (1:100 clone 145-2Cll, eBioscience), Ly-6G/Gr-1 (1:100 clone RB6-8C5, eBioscience), CDllb/Mac-1 (clone Ml/70, BD Pharmingen), CD45R/B220 (clone RA3-6B2, BD Pharmingen), Feε Rl α (clone MAR-1, eBioscience), CD8a (1:100 clone 53-6.7, BD Pharmingen), CDllc (1:100 clone N418, eBioscience); TER-119 (1:100 clone Ter119, BD Pharmingen), anti-c-Kit (1:100 PerCP- Cy5.5, clone 2B8, eBioscience), anti-Sca-1 (1:100 PE-Cy7, clone D7, eBioscience); anti-CD48 (1:100 FITC, clone HM48-1, BioLegend), anti-CD41 (1:100 FITC, clone MWReg30, BD Pharmigen), anti-CD150 (1:100 APC, clone TC15-12F12.2, BioLegend), anti-SSEA1 antibody conjugated to Alexa Fluor 647 (1:100, catalogue no. MC-480; BioLegend) Anti-phospho-Ser139-Histone H2A.X (1:1,000, catalogue no. 05-636; Merk Millipore), anti-phospho- Ser15-TP53 (1:500, catalogue no. D4S1H; Cell Signalling Technology), anti-cleaved caspase-3 (1:300, catalogue no. D175; Cell Signalling Technology), anti-glial acidic filament protein (GFAP) (1:500, catalogue no. Z0334; Dako), anti-MAC2 (1:2,000, catalogue no. 125402; BioLegend), anti-Ki67 (1:100, catalogue no. M3062; Spring Science), Anti-p21 (1:500,[HUGO291], ab107099, Abcam), anti-PCNA (1:1000, PCNA (PC10) Mouse mAb Cell signalling catalogue no. 2586).

Validation

anti-SSEA1: https://www.biolegend.com/fr-lu/products/alexa-fluor-647-anti-mouse-human-cd15-ssea-1-antibody-4819?

phospho-Ser139-Histone H2A.X: https://www.merckmillipore.com/GB/en/product/Anti-phospho-Histone-H2A.X-Ser139-Antibody-clone-JBW301,MM_NF-05-636

phospho- Ser15-TP53 : https://www.cellsignal.com/products/primary-antibodies/phospho-p53-ser15-d4s1h-rabbit-mab/12571

cleaved caspase-3: https://www.cellsignal.com/products/primary-antibodies/cleaved-caspase-3-asp175-d3e9-rabbit-mab/9579

glial acidic filament protein (GFAP): https://www.agilent.com/store/productDetail.jsp?catalogId=Z033401-2

MAC2: https://www.biolegend.com/en-us/products/purified-anti-mouse-human-mac-2-galectin-3-antibody-4935

Ki67: https://www.nordiqc.org/protocol_view.php?id=1411

p21: https://www.abcam.com/products/primary-antibodies/p21-antibody-hugo291-ab107099.html

PCNA: https://www.cellsignal.com/products/primary-antibodies/pcna-pc10-mouse-mab/2586

CD4, CD3e, Ly-6G/Gr-1, CDllb/Mac-1, CD45R/B220, Feε Rl α, CD8a, CDllc ; TER-119, c-Kit, Sca-1 CD48, CD41, CD150: This panel has previously been validated PMID: 22922648, 25707806, 23827712, 15989959

Animals and other research organisms

Policy information about studies involving animals; ARRIVE guidelines recommended for reporting animal research, and Sex and Gender in Research

Laboratory animals

All animal experimentation was undertaken in this study were approved by the Medical Research Council's Laboratory of Molecular Biology animal welfare and ethical review body and the UK Home Office under the Animal (Scientific Procedures) Act 1986 (licence number PP6752216). All mice used in this study were maintained under pathogen-free conditions and housed in individually ventilated racks (GM500; Techniplast) on Lignocel FS-15 spruce bedding (IPS) and provided with environmental enrichment (chew stick, fun tunnel and Enviro-Dri nesting (LBS)). Animals were maintained at 19-23ºC with light from 07:00 to 19:00 and fed Dietex CRM pellets (SDS) ad libitum. No were wild and no field-collected samples were used in this study. Unless otherwise stated, mice were generated on a C57BL/6 x 129S4S6/Sv F1 genetic hybrid background. Samples were collected from adult mice at various stages as stated in the text. The investigators were blinded to the genotypes of animals throughout the study and data were acquired on the basis of identification numbers only. Ercc1tm1a(KOMP)Wtsi (MGID: 4362172), Trp53tm1Brd (MGID: 1857590) and GOF18-GFP (Tg(Pou5f1-EGFP)11Ymat) (MGID: 6148237) have been described previously.

Wild animals

No wild animals were used

Reporting on sex

Reproductive issues were examined therefore males and females were treated separately as detailed in the text

Field-collected samples	This study did not involve field-collected samples.
Ethics oversight	All animal experiments undertaken in this study were approved by the Medical Research Council's Laboratory of Molecular Biology animal welfare and ethical review body and the UK Home Office under the Animal (Scientific Procedures) Act 1986 (license no. PP6752216).

Note that full information on the approval of the study protocol must also be provided in the manuscript.

Flow Cytometry

Plots

Confirm that:

☒ The axis labels state the marker and fluorochrome used (e.g. CD4-FITC).

☒ The axis scales are clearly visible. Include numbers along axes only for bottom left plot of group (a 'group' is an analysis of identical markers).

☒ All plots are contour plots with outliers or pseudocolor plots.

☒ A numerical value for number of cells or percentage (with statistics) is provided.

Methodology

Sample preparation	Fresh livers and kidneys were isolated from age-matched mice and stored in ice-cold PBS on ice. Subsequently, 250 mg of tissue was passed through a 70 um cell strainer and washed twice in buffer LA (250 mM sucrose, 5 mM MgCl2, 10 mM Tris-HCl, pH 7.4). Cells were resuspended washed twice in 1 mL of buffer LB (2 M sucrose, 1 mM MgCl2, 10 mM Tris-HCl, pH 7.4) and centrifuged at 16,000 x g at 4ºC for 30 minutes. Subsequently, nuclei were resuspended in 1 mL buffer LA and transferred to a fresh 15 mL Falcon tube and fixed in 70% ethanol overnight at -20ºC. Nuclei were resuspended in staining buffer (PBS supplemented with propidium iodide (40 ug.mL-1, Sigma) and Ribonuclease A from bovine pancreas (100 ⍰ g.mL-1, Sigma)) and incubated at room temperature for 20 minutes. Samples were then immediately run on an LSRII FACS analyser (BD Pharmingen) For PGC quantification, the genital ridge of developing embryos carrying the GOF18-GFP reporter were isolated as described above and placed into 150 ⍰L trypsin solution pre-warmed to 37ºC and incubated for 10 minutes at 37ºC in a water bath. Subsequently, 1 uL Benzonase (99% purity, Millipore) was added and the sample disaggregated by gentle pipetting and incubated for a further 5 minutes at 37ºC in a water bath. Trypsin was inactivated by adding 1 mL of PBS/5% v/v FCS and samples centrifuged at 1,100 g.for 10 minutes. The supernatant was discarded and the cell pellet resuspended in 100 uL of anti-SSEA1 antibody conjugated to Alexa Fluor 647 (1:100, catalogue no. MC-480; BioLegend) and incubated at room temperature for 10 minutes. Samples were then diluted by adding 300 uL PBS/5% v/v FCS and immediately run on an ECLIPSE analyser (Sony Biotechnology)<br>For HSC quantification, bone marrow cells were harvested from the femurs and tibiae of 6-to-8-week-old adult mice in staining buffer (PBS/2.5% v/v FCS) and filtered through a 70 ⍰m cell strainer. Erythrocytes were lysed by resuspending samples in 10 mL of red cell lysis buffer (MACS Miltenyi Biotec) for 10 minutes at room temperature. Cells were the resuspended in 1 mL of staining buffer and the number of nucleated cells quantified by diluting 10 ⍰L of cell suspension in 990 ⍰L 3% acetic acid solution (StemCell Technologies) and quantified using a Vi-Cell XR cell viability counter (Beckman Coulter). Subsequently, 10 x 106 nucleated bone marrow cells were stained in 200 ⍰L of staining solution containing the following antibodies; FITC-conjugated lineage cocktail (anti-CD4 (clone H129.12; BD Pharmingen), anti-CD3e (clone 145-2 C11, eBioscience), anti-Ly-6G/Gr-1 (clone RB6-8Cs, eBioscience), anti-CD11b/Mac-1 (clone M1/70, BD Pharmingen), anti-CD45R/B220 (clone RA3-6B2, BD Pharmingen), anti-Fc⍰ R1⍰ (clone MAR-1, eBioscience), anti-CD8a (clone 53-6/7, BD Pharmingen), anti-CD11c (clone N418, eBioscience), anti-TER-119 (clone Ter119, BD Pharmingen) and anti-CD41 (clone MWReg30, BD Pharmingen); anti- c-Kit (PerCP-Cy5.5, clone 2B8, eBioscience), anti-Sca-1 (PE-Cy7, clone D7, eBioscience), anti-CD150 (PE, clone TC15-12F12.2, eBioscience), and anti-CD48 (biotin, clone HM48.1, BioLegend). Samples were incubated with the primary antibodies for 15 minutes at room temperature and washed in 1 mL of staining buffer. Samples were then resuspended in 200 ⍰L of staining buffer containing streptavidin-BV421 and incubated for 15 minutes at room temperature. Finally, samples were washed in 1 mL staining solution and resuspended in 400 ⍰L of staining buffer and immediately run on an LSRII FACS analyser (BD) and the data analysed using FlowJo v.10.1r5 (FlowJo LLC). HSPCs were defined as lineage-, CD41-, Sca-1+, and c-Kit+ and HSCs define as lineage-, CD41-, Sca-1+, c-Kit+, CD48- and CD150+.
Instrument	LSRII analyzer (BD Biosciences)
Software	Data was collected using FACSDiva (BD) and processed using FlowJo v10.1r5.
Cell population abundance	N/A no samples were sorted
Gating strategy	For PGC quantification, cells were gated using FSC/SSC and PGCs defined as (APC-SSEA+GFP+)<br>HSPCs were defined as lineage-, CD41-, Sca-1+, and c-Kit+ and HSCs define as lineage-, CD41-, Sca-1+, c-Kit+, CD48- and CD150+.<br>As shown in Supplementary Figure 11.

☒ Tick this box to confirm that a figure exemplifying the gating strategy is provided in the Supplementary Information.
